# Immediate Effects of Dry Needling on Central Pain Processing and Skin Conductance in Patients with Chronic Nonspecific Neck Pain: A Randomized Controlled Trial

**DOI:** 10.3390/jcm11226616

**Published:** 2022-11-08

**Authors:** Marcos José Navarro-Santana, Juan Antonio Valera-Calero, Guillermo Romanos-Castillo, Victor C. Hernández-González, César Fernández-de-las-Peñas, Ibai López-de-Uralde-Villanueva, Gustavo Plaza-Manzano

**Affiliations:** 1 Faculty of Health, Universidad Católica de Ávila, C/Canteros, s/n, 05005 Ávila, Spain; 2 VALTRADOFI Research Group, Department of Physiotherapy, Faculty of Health, Universidad Camilo José Cela, Villanueva de la Cañada, Villanueva de la Cañada, 28692 Madrid, Spain; 3 Department of Physiotherapy, Faculty of Health, Universidad Camilo José Cela, Villanueva de la Cañada, 28692 Madrid, Spain; 4 Department of Physical Therapy, Occupational Therapy, Physical Medicine and Rehabilitation, Universidad Rey Juan Carlos, 28922 Alcorcón, Spain; 5 Cátedra Institucional en Docencia, Clínica e Investigación en Fisioterapia: Terapia Manual, Punción Seca y Ejercicio Terapéutico, Universidad Rey Juan Carlos, 28922 Alcorcón, Spain; 6 Department of Radiology, Rehabilitation and Physiotherapy, Universidad Complutense de Madrid, 28040 Madrid, Spain; 7 Grupo InPhysio, Instituto de Investigación Sanitaria San Carlos (IdISSC), 28040 Madrid, Spain

**Keywords:** dry needling, sham needling, neck pain, conditioned pain modulation, skin conductance, temporal summation, pressure pain thresholds

## Abstract

Although current evidence supports the use of dry needling for improving some clinical outcomes in people with neck pain, no previous research explored the effects of dry needling on the central processing of pain and autonomic nervous system in this population. Therefore, this clinical trial aimed to compare the effects of real and sham dry needling on autonomic nervous system function, pain processing as well as clinical and psychological variables in patients with chronic nonspecific neck pain. A double-blinded randomized clinical trial including 60 patients with neck pain was conducted. Patients were randomized to the real needling (*n* = 30) or sham needling (*n* = 30) group. Skin conductance (SC), pressure pain thresholds (PPTs), temporal summation (TS), conditioned pain modulation (CPM) as well as pain intensity, related-disability, catastrophism, and kinesiophobia levels were assessed by an assessor blinded to the allocation intervention. The results did not find significant group * time interactions for most outcomes, except for the global percentage of change of SC values (mean: F = 35.90, *p* < 0.001, $\eta_{p}^{2}$ = 0.459; minimum: F = 33.99, *p* = 0.839, $\eta_{p}^{2}$ = 0.371; maximum: F = 24.71, *p* < 0.001, $\eta_{p}^{2}$ = 0.037) and PPTs at C5-C6 joint in the same side of needling (F = 9.982; *p* = 0.003; = 0.147), in favor of the dry needling group. Although the proportion of subjects experiencing moderate to large self-perceived improvement after the intervention was significantly higher (X2 = 8.297; *p* = 0.004) within the dry needling group (*n* = 18, 60%) than in the sham needling group (*n* = 7, 23.3%), both groups experienced similar improvements in clinical and psychological variables. Our results suggested that dry needling applied to patients with chronic nonspecific neck pain produced an immediate decrease in mechanical hyperalgesia at local sites and produced an increase in skin conductance as compared with sham needling. No changes in central pain processing were observed. A single session of sham or real dry needling was similarly effective for decreasing related disability, pain intensity, catastrophism, and kinesiophobia levels. Further studies are needed to better understand the clinical implications of autonomic nervous system activation on central sensitization and pain processing in the long-term after the application of dry needling.

## 1. Introduction

Neck pain is a common musculoskeletal complaint which affects the quality of life of individuals. In fact, neck pain is ranked in the fourth position as a condition causing the greater number of years lived with disability [1]. Neck pain is estimated to affect up to 20% of adults worldwide, with a lifetime prevalence of 70% in the general population [2].

One common therapeutic strategy in clinical practice for the management of musculoskeletal neck pain is dry needling. Dry needling consists of a skilled intervention which uses a thin filiform needle (as those used in acupuncture) to penetrate the skin and stimulate underlying myofascial trigger points, muscular, and connective tissues for the management of neuromusculoskeletal pain and movement impairments [3]. Low to moderate evidence supports the use of dry needling as a potential effective choice for the management of neck pain, at least at short and mid-term follow-ups [4]. One important difference between Eastern medicine-based practice of acupuncture and Western medicine-based dry needling is the fact that dry needling targets myofascial trigger points (TrPs). Consistent evidence associates the presence of TrPs, particularly in the upper trapezius and scapulae elevator muscles, in individuals with neck pain [5].

Current evidence supports that physical therapy interventions are able to modulate central pain processing (by decreasing temporal summation and increasing conditioning pain modulation) [6] and to induce a sympatho-excitatory effect (i.e., increase in skin conductance and a decrease in skin temperature) [7]. Since dry needling induces analgesic responses with minimal effects on muscle stiffness [8], similar neurophysiological mechanisms were also proposed for this intervention [9]. In fact, Fernández-de-las-Peñas and Nijs [9] proposed a model where peripheral and central mechanisms are involved at the same time in the therapeutic effect of dry needling. However, these potential mechanisms have not been fully demonstrated and remain poorly understood.

Some studies have investigated whether dry needling induces changes in the central pain processing [10] and autonomic nervous system [11]. Vervullens et al. [10] observed that a single dry needling session had no effect on central pain processing as compared to sham needling in a sample of individuals with knee osteoarthritis. Lázaro-Navas et al. [11] found that a single session of dry needling produced an immediate activation in the sympathetic nervous system (as assessed by heart rate variability) in healthy individuals. To the best of the authors’ knowledge, no previous research has further explored the effects of dry needling on the central pain processing and autonomic nervous system in people with chronic nonspecific neck pain [12]. Since treatment strategies targeting the cervical spine seem to have higher endocrine responses as compared with other locations (specially in chronic pain populations) [13,14,15], we used chronic neck pain as the model for the current study.

Accordingly, the main objective of this clinical trial was to determine the immediate effects of a single dry needling session within autonomic nervous system activity (i.e., skin conductance) and central pain processing (i.e., pressure pain sensitivity, temporal summation, and conditioned pain modulation) in patients with chronic nonspecific neck pain as compared with sham dry needling. As a secondary objective, we analyzed whether real or sham dry needling induced changes in clinical (i.e., pain intensity, related-disability) and psychological (i.e., kinesiophobia, catastrophism) variables one-week after.

## 2. Methods

### 2.1. Study Design

A randomized, placebo-controlled, double-blinded clinical trial comparing the effects of a single session of real dry needling versus sham dry needling in patients with chronic nonspecific neck pain was conducted. This trial followed the CONsolidated Standards Of Reporting Trials (CONSORT) guidelines for pragmatic clinical trials [16] and the Enhancing the QUAlity and Transparency Of health Research (EQUATOR) guidelines [17]. Since humans with neck pain were involved in this experimental study, we respected the rights of the participants according with the Declaration of Helsinki and the study design was evaluated and approved by the Institutional Ethics Committee of Universidad de Alcalá, Spain (CEIM/HU/2015/18). In addition, the study protocol was prospectively registered in Clinicaltrials.org on 26 May 2022 (NCT05391568).

Sociodemographic (i.e., caffeine and tobacco consumption, age, sex, weight, height, and body mass index), clinical (i.e., pain intensity, pain-related disability, global rating of change), autonomic nervous system activity (i.e., skin conductance), psychophysical (i.e., pressure pain thresholds, conditioned pain modulation, and temporal summation), and psychological (i.e., fear-avoidance and catastrophizing behavior) variables were evaluated. Skin conductance and psychophysical variables were collected before (baseline) and immediately after the needling intervention. Clinical and psychological variables were collected before (baseline) and one-week after each intervention. All outcomes were evaluated by an assessor blinded to the treatment allocation of the subjects.

### 2.2. Participants

Voluntary participants were recruited from a third-party private physiotherapy clinic located in Madrid (Spain) during June 2022. Potential participants were firstly identified by their clinical history and contacted by mail. To be eligible, patients had to: (1) be adults with an age between 18 and 65 years old; (2) suffer chronic nonspecific neck pain symptoms for at least 3 months of duration [18]; (3) presenting a pain intensity of >3/10 points in the numeric pain rating scale (NPRS, 0–10); and (4) read, understand, and sign the informed consent form prior to their participation. Those participants with any of the following criteria were excluded: (1) neurological symptoms or signs compatible with cervical radiculopathy or myelopathy; (2) cervical trauma (e.g., whiplash); (3) systemic diseases; (4) pregnancy; (5) psychiatric conditions; or (6) contraindication to dry needling (e.g., fear of needles or being under anticoagulants treatment). Participants were advised to avoid any painkiller intake during the study period, and caffeine or tobacco consumption before the day of assessment.

### 2.3. Sample Size Calculation

The minimum sample size required for this study was calculated using the G*Power software v.3.1.6. (Heinrich, Heine University, Düsseldorf, Germany) for Mac OS. The calculus was conducted using an a priori analysis with a F test ANOVA for repeated measures within factors setting a 5% of significance level (α = 0.05), 80% of statistical power, 2 groups, and 4 repeated measurements for our primary outcome (skin conductance). The effect size (f = 0.143; −21.50 ± 1.99 for real dry needling and −10.36 ± 1.96 for sham dry needling), correlation among repeated measures (0.6), and nonsphericity correction (ϵ = 1) were obtained from a previous pilot study with 15 participants per group. Based on these parameters, a minimum sample size of 56 participants (28 participants per group) could be considered appropriate.

### 2.4. Randomization and Blinding

Concealed allocation was performed with block randomization for sixty patients and two groups using computer-generation before the study started. Both participants and assessors were blinded to the treatment allocation group.

### 2.5. Primary Outcome: Skin Conductance

Autonomic nervous system activity was measured through the skin conductance (SC) while applying either dry needling or sham dry needling with the Mindfield eSense Skin Response hardware and software for iPad OS as it is shown to be a valid tool for assessing psychophysiological reactivity [19]. Skin conductance measurement is a popular method (specially in psychophysiology research) for assessing the sympathetic nervous system activity since variations of the skin electrical conductivity due to sweat secretions are relatively fast and previous evidence demonstrated how it reflects the sympathetic nervous system responses [20].

**Figure 1:**
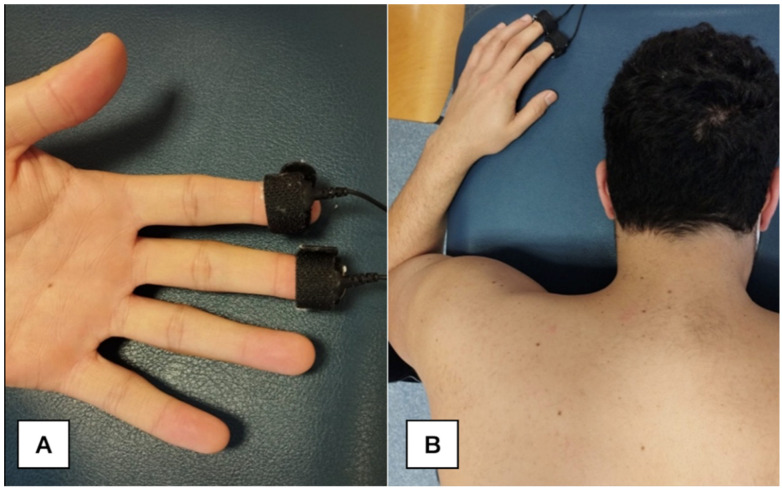
Skin conductance setting (**A**). Participant positioning (**B**).

The skin conductance was expressed in microSiemens and, since medical-related sounds provoke an increased skin conductance level [21], skin conductance was collected with the patient in prone position (Figure 1) at the following periods: baseline (the last minute before verbal warning); verbal warning (time of verbal warning to perform real or sham needling); during the intervention (time lasting real or sham needling); and post-intervention (the last minute, 5 min after intervention). We calculated mean, maximum, and minimum values of skin conductance.

Due to the nature of the assessment, the assessor evaluating skin conductance was not involved in any other measurement. Blinding of the assessor was maintained until the end of each intervention.

### 2.6. Secondary Outcomes

#### 2.6.1. Clinical Variables

Neck pain intensity during the previous week was measured on a 10-point numeric pain rating scale (NPRS). In this scale, patients were asked to rate from 0 (no pain) to 10 (worst pain) their mean pain intensity, as the NPRS has been shown to be reliable (ICC 0.76 95%CI, 0.51–0.87) and valid for assessment of nonspecific neck pain and with a minimum clinically important difference (MCID) of 1.5 points [22].

The Northwick Park Neck Pain Questionnaire (NPQ) was used for assessing the pain-related disability based on 9 items assessing different aspects of the patient’s pain and limitations in the daily activities experience [23] since this tool has shown a good test-retest reliability (ICC 0.63, 95%CI 0.44–0.76) [24]. Scores are ranges between 0 and 1 and are calculated using the following formula NPQ (score/total score of each question answered). Final scores are expressed as percentage (%),with 0 being the complete absence of disability and pain and 100, the worst disability and pain [23,24].

The Global Rating of Change Scale (GRoC) was also assessed. Each patient completed a GRoC as described by Jaeschke et al. [24]. Patients were asked to provide feedback about their perceived improvement after the intervention using a 15-point Likert scale ranging −7 (a very great deal worse) to +7 (a very great deal better), with 0 being “no changes at all”. Scores +4 and +5 indicate moderate changes, whereas scores of +6 and +7 indicate large changes [25].

#### 2.6.2. Psychological Variables

The Pain Catastrophizing Scale (PCS) is a 12-item questionnaire answered in a 5-point Likert scale (where 0 means “never” and 4 means “always”) commonly used for assessing different catastrophizing behaviors against pain including rumination (constant worry and inability to inhibit thoughts related to pain), magnification (exaggeration of unpleasantness of painful situations and expectations of negative consequences), and despair (inability to face pain) [26]. This instrument was used as demonstrated to be a valid instrument with an appropriate internal consistency (Cronbach alpha 0.79) and a good test-retest reliability (ICC 0.84) [27].

The Tampa Scale for Kinesiophobia (TSK-11) was used to evaluate whether the patients exhibited behaviors related with fear of movement and avoidance. The TSK-11 is composed by 17 items answered in a 4-point Likert scale of how much they agree with each item, with 1 being “complete disagreement” and 4 “complete agreement”. Therefore, the total score can range between 11 and 44 points, where greater scores are associated with greater fear avoidance [28]. TheTSK-11 was used as it has demonstrated an appropriate internal consistency (Cronbach alpha 0.79) [29].

#### 2.6.3. Psychophysical Variables

Pressure pain thresholds (PPTs), temporal summation (TS), and conditioned pain modulation (CPM) were tested for evaluating central pain processing. These outcomes were tested with a 10-min resting period between.

First, PPTs were measured using a digital algometer (Force Dial FDK 20 Wagner algometer) with a round surface area of 1 cm^2^, applying a perpendicular pressure to the skin at an increasing rate of 1 kg/cm^2^/s as this procedure has shown good reliability (ICC 0.91) [30]. PPTs over the C5-C6 zygapophyseal joints (in prone), the dorsal aspect of the midpoint between the base of the nail and the interphalangeal joint of the index finger (seated) and the tibialis anterior muscle (in supine) were bilaterally assessed. The mean of 3 trials (with a 30-s resting period) were calculated to be used for the analyses. The means of PPTs on the hand and the tibialis anterior were used in the analyses for identifying widespread pressure sensitivity.

Second, the pressure stimulus for TS was determined. For this purpose, a pressure over the second finger of the hand was performed until the patient experienced a sensation of pain of 6/10 points on a NPRS, increasing the pressure at a rate of approximately 1 kg/s [31]. Then, using this targeted pressure, 10 stimuli were applied to register their perceived pain intensity at the 1st, 5th, and 10th stimuli [32]. The difference between 1st and last stimuli (10th stimuli) was calculated to determine TS. An increase in pain perception (NPRS, 0–10) at the 10th stimuli in relation to the 1st stimuli represents TS.

Lastly, conditioned pain modulation (CPM) was tested using a cuff pressor in the lower limb (choosing the limb in the treatment’s opposite side) since this procedure has demonstrated good to excellent intersession reliability (ICC > 0.6–0.90) [33]. Participants were placed in a sitting position and were asked to raise their limb and rest it on a chair. The pressure cuff was placed in the ankle at a pressure of 250–260 mmHg. Then, patients were asked to perform ankle dorsal flexions until reaching a pain sensation of 6 points in the NPRS [34]. Again, PPT and TS over the second finger were evaluated immediately after the application the cuff inflation to evaluate activation of CPM. CPM was assessed by calculating the difference in PPT before and after cuff inflation. An impaired CPM is operationally defined as no change or a negative change in PPTs, taken directly following termination of the conditioning stimulus (cuff pressor) [35]. In addition, we also evaluated activation of CPM by calculating the difference in TS before and after cuff inflation considering the 10st stimuli (negative values reveal an increase of pain intensity).

Verbal advises were not evaluated on psychophysical variables since a recent study has found that verbal suggestion did not influence neither the perception of pain perceived during the needling procedure nor TS or CPM in patients with latent TrPs in the upper trapezius muscle [36].

### 2.7. Interventions

A recent study observed that the effects of dry needling are higher when applied on active TrPs as compared when applied on a latent TrP or a non-TrP area in patients with neck pain [37]. Accordingly, each group received the needling intervention (sham or real) over an active TrP located in the upper trapezius muscle by an experienced physical therapist with more than 15 years of clinical and research experience in musculoskeletal pain. In the case of more than one active TrP on the same side or bilateral TrPs, the intervention targeted the most symptomatic side (e.g., reproducing familiar symptoms or the most mechanosensitive). Once the TrP was located, the overlying skin was cleaned with alcohol. All the subjects were systematically notified (previous to the intervention) with the following verbal message “I am going to perform a dry needling intervention”.

**Figure 2:**
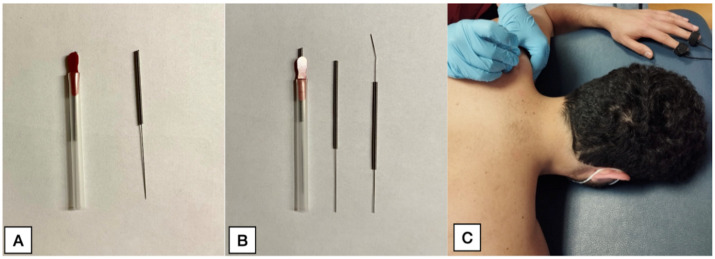
Real needles (**A**). Sham needles (**B**). Application of either needling intervention (**C**).

For patients receiving real dry needling, one disposable stainless-steel needle of 0.32 mm × 25 mm (Agupunt, Barcelona, Spain, Figure 2A) was inserted through the skin targeting the MTrP and using the fast-in and fast-out technique described by Hong [38] for 30 s). The number of local twitch responses were registered by the therapist and intensity of pain during the technique (NPRS, 0–10 points). Upon removal of the needle, the area was compressed firmly with a cotton ball for approximately 1 min since it is encouraged during clinical practice and has been demonstrated to reduce post-needling soreness intensity and duration [39].

For participants receiving sham needling, the telescopic Park’s sham device was used (Dongbang Medical Co., Ltd., Sungnam, South Korea). The handle was tapped briskly, but the (blunted) needle tip did not penetrate and break the skin (Figure 2B). The sham needle retracted within the guide tube and was pressed against the skin, simulating the same procedure described for real dry needling [8]. The intensity of pain and NPRS during the technique was registered. Finally, after finishing the intervention, the area was also compressed firmly with a cotton ball for approximately 1 min to avoid bias.

### 2.8. Statistical Analysis

Statistical analyses were performed using SPSS v.27 software for MacOS and were conducted according to the intention to treat analysis principle. The Kolmogorov–Smirnov test showed a normal distribution of the data (*p* > 0.05), accordingly, means, standard deviations, and/or 95% confidence intervals were calculated for each quantitative variable. Categorical data were described by using frequency analyses. Baseline demographic and outcomes were compared between groups using independent Student’s *t*-tests for continuous data and *χ*2 tests of independence for categorical data. For the primary outcome (skin conductance), a multivariate general lineal model with the time-point (pre-intervention, verbal warning, intervention, and post-intervention) and group (real or sham needling) as fix factors was conducted. For the secondary measures, a repeated measure analysis of variance (ANOVA) with time-point (before and after the intervention for psychophysical outcomes; before and one-week after intervention for clinical or psychological outcomes) as the withing-subject factor and group (real or sham needling) as the within-subjects factor was conducted. Within-group and between-groups comparisons were analyzed using Student’s t-tests for independent samples as post hoc analyses. Due to the use of multiple comparisons, the Bonferroni correction was applied. Accordingly, *p* values were assumed to be significant at <0.0125 (0.05/4). Finally, the effect size was estimated using the *η_p_*^2^ if significant. Partial eta squared was used instead of Cohen’s d since this statistical estimate is recommended in ANOVA models [40]. An effect size of 0.01 was considered small, 0.06 medium, and 0.14 large.

## 3. Results

**Figure 3:**
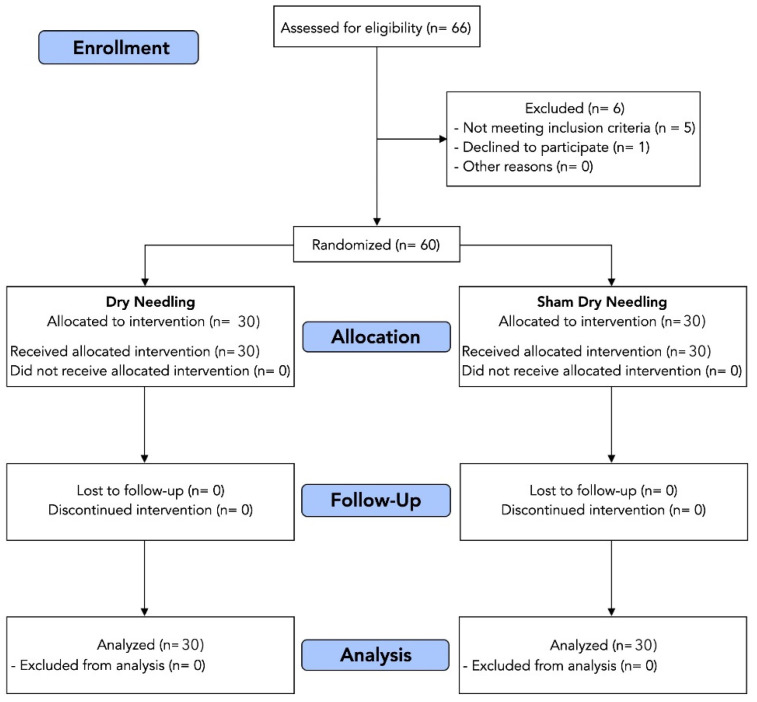
Flow diagram of patients throughout the course of the study.

From a total of 66 consecutive patients with neck pain symptoms who were initially screened for eligibility, 60 (90%) satisfied the inclusion criteria, agreed to participate, and were randomly allocated into real dry needling (*n* = 30) or sham dry needling (*n* = 30) group. Randomization resulted in similar baseline characteristics for all variables (Table 1). Figure 3 illustrates the flow diagram of patients throughout the course of the study. None of the participants in either group abandoned or were lost.

### 3.1. Skin Conductance

Table 2 details changes of skin conductance after real or sham needling. The results did not reveal any significant group * time interaction (mean: F = 1.709, *p* = 0.192, $\eta_{p}^{2}$ = 0.029; minimum: F = 0.145, *p* = 0.839, $\eta_{p}^{2}$ = 0.002; maximum: F = 2.207, *p* = 0.129, $\eta_{p}^{2}$ = 0.037). A significant main effect of time was seen (mean: F = 49.21, *p* < 0.001, $\eta_{p}^{2}$ = 0.459; minimum: F = 47.940, *p* < 0.001, $\eta_{p}^{2}$ = 0.453; maximum: F = 49.30, *p* < 0.001, $\eta_{p}^{2}$ = 0.458). Overall, both groups exhibited similar changes on skin conductance throughout the study. The pos hoc analyses revealed significant group * time interactions of the global percentage of change of skin conductance values (mean: F = 35.90, *p* < 0.001, $\eta_{p}^{2}$ = 0.459; minimum: F = 33.99, *p* = 0.839, $\eta_{p}^{2}$ = 0.371; maximum: F = 24.71, *p* < 0.001, $\eta_{p}^{2}$ = 0.037).

### 3.2. Clinical and Psychological Variables

The repeated measured ANOVA revealed no significant group * time interaction for any clinical (pain: F = 2.700; *p* = 0.253; $\eta_{p}^{2}$ = 0.023; related-disability: F = 2.813; *p* = 0.219; $\eta_{p}^{2}$ = 0.009) or psychological (PCS F = 0.163; *p* = 0.688; $\eta_{p}^{2}$ = 0.003; TSK-11: F = 0.308; *p* = 0.581; $\eta_{p}^{2}$ = 0.005) outcome. In fact, significant main effect of time was observed: pain (F = 61.347; *p* < 0.001; $\eta_{p}^{2}$ = 0.514); related-disability (F = 46.801; *p* < 0.001; $\eta_{p}^{2}$ = 0.447), PCS (F = 17.906; *p* < 0.001; $\eta_{p}^{2}$ = 0.236), or TSK-11 (F = 5.577; *p* = 0.022; $\eta_{p}^{2}$ = 0.088). Both groups experienced similar improvements in these outcomes (Table 3).

The proportion of subjects experiencing moderate to large self-perceived improvement (at least scored +4) as assessed by the GRoC one-week after the intervention was significantly higher (*X*^2^ = 8.297; *p* = 0.004) within the dry needling group (*n* = 18, 60%) as compared to the sham needling group (*n* = 7, 23.3%).

### 3.3. Psychophysical Variables

The ANOVA revealed a significant group * time interaction for PPTs on the C5-C6 joint in the same side of needling (F = 9.982; *p* = 0.003; $\eta_{p}^{2}$ = 0.147), but not for PPTS on the remaining points (C5-C6 joint contra-lateral side: F = 2.158; *p* = 0.147; $\eta_{p}^{2}$ = 0.036; second finger: F = 0.034; *p* = 0.854; $\eta_{p}^{2}$ = 0.001; tibialis anterior: F = 0.020; *p* = 0.887; $\eta_{p}^{2}$ < 0.001): individuals receiving real dry needling experienced an increase in PPT (hypoalgesic effect) over the C5-C6 joint of the affected side when compared with those receiving sham needling (Table 4). No significant main effect of time was either observed (C5-C6 joint same side: F = 1.391; *p* = 0.243; $\eta_{p}^{2}$ = 0.023; C5-C6 contra-lateral side: F = 2.363; *p* = 0.130; $\eta_{p}^{2}$ = 0.039; second finger: F = 0.780; *p* = 0.381; $\eta_{p}^{2}$ = 0.013; tibialis anterior: F = 1.826; *p* = 0.182; $\eta_{p}^{2}$ = 0.031).

Similarly, the repeated measure ANOVA did not reveal any significant group * time interaction for TS (F = 0.111, *p* = 0.740; $\eta_{p}^{2}$ = 0.002); PPT after CPM (F = 0.050, *p* = 0.825; $\eta_{p}^{2}$ = 0.001); and TS after CPM (F = 0.346, *p* = 0.559; $\eta_{p}^{2}$ = 0.006). No significant main effect for time was either found (TS: F = 1.774; *p* = 0.188; $\eta_{p}^{2}$ = 0.030; PPT after CPM: F = 0.103; *p* = 0.749; $\eta_{p}^{2}$ = 0.002; TS after CPM: F = 0.001; *p* = 0.973; $\eta_{p}^{2}$ < 0.001). Table 4 summarizes the effects of real or sham dry needling on central pain processing outcomes.

## 4. Discussion

### 4.1. Findings

The main aim of this clinical trial was to determine the immediate effects of a single dry needling session in autonomic nervous system function and central pain processing in patients with chronic nonspecific neck pain in comparison with sham dry needling. The results of this study are consistent with previous research showing that dry needling had no central effects despite local PPT improvements in structures close to the intervention area. In addition, dry needling had no effect on neural excitability (measured by TS) nor the diffuse noxious inhibitory control (measured by CPM). Maybe one of the most interesting findings is the fact that dry needling seems to induce autonomic nervous system responses (measured by SC), since we identified greater percentages of change (but not absolute values) of skin conductance when compared with sham needling. Finally, a single session of dry needling was equally effective as sham dry needling for reducing pain related-disability, but not for improving pain intensity, catastrophism, or kinesiophobia levels. These outcomes may need a larger number of sessions to show significant psychophysical responses.

### 4.2. Changes in Skin Conductance

The results of the current trial on skin conductance are consistent with the findings reported by Lázaro-Navas et al. [11] who reported whether real and sham dry needling produces an immediate activation in the sympathetic nervous system. This similarity between real and sham dry needling might be due to the lack of verbal warning applied before the intervention. Our results analyzing skin conductance changes after verbal warning and before the interventions demonstrated significant changes in this time frame. Although verbal warning is enough to produce a change in skin conductance, the change before verbal warning and after the intervention was greater in the dry needling group (34% vs. 91.5%), supporting a potential real effect of dry needling.

Although a previous study using acupuncture found no associations between autonomic nervous system activity and the analgesic response [41], Lázaro-Navas et al. [11] reported a positive correlation between mechanical hyperalgesia at remotes locations and the skin temperature. The correlation of autonomic nervous system activation and analgesia is still unknown, and further research is needed.

### 4.3. Changes in Central Pain Processing

Our results are consistent with previous studies assessing the effect of dry needling on the nociceptive pain processing. For instance, Sanchez-Romero et al. [36] did not find changes on TS and CPM in healthy participants after DN of latent TrP in the upper trapezius muscle. In addition, Vernulles et al. [10] analyzed the effect of real dry needling in patients with knee osteoarthritis and also found no changes on TS nor CPM. However, the CPM in remote locations showed significant differences between real dry needling and sham needling. In contrast with their results, we did not find any change on CPM in a remote location. In fact, controversial evidence about immediate changes in remotes areas after dry needling in patients with chronic nonspecific neck pain is currently under debate [8,42,43]. Stieven et al. [42] found no PTT changes in remote locations in accordance with our findings. In contrast, Mejuto-Vázquez et al. [43] and Valera-Calero et al. [8] demonstrated changes in PPTs at distal locations in patients with acute mechanical neck pain after a dry needling intervention.

Regarding the local effects of dry needling, our results suggested an immediate local PPT increase, suggesting a local hypoalgesic effect. A previous metanalysis showed a significant immediate effect of dry needling compared with inactive controls or sham dry needling [4]. Despite these results suggesting that dry needling has a positive effect in local mechanical hyperalgesia, remote areas showed no changes. Therefore, the effect on central pain processing may be due to the cessation of peripheral sensitization and it cannot be detected immediately.

### 4.4. Clinical Implications

Our results suggest a limited effect of dry needling on central pain processing in people with chronic neck pain since just local PPT changes were found. Chronic pain conditions are characterized by lower PPT in remote sites, indicating widespread hyperalgesia and central sensitization [44,45] and it is possible that central sensitization processes cannot be modified with just a single dry needling intervention. The isolate use of dry needling during clinical practice is uncommon and a combination of dry needling with other interventions could possibly have a greater effect in pain processing. Fernandez-de-las-Peñas et al. [46], in a recent metanalysis, found significant changes between combined treatments in comparison with isolated interventions, whereas Navarro-Santana et al. [4] did not find changes between the isolated use of dry needling with other isolated manual therapy interventions. Additionally, we applied a single session of dry needling, which does not represent common clinical practice. It is possible that consecutive application of a larger number of sessions could lead to different results. In fact, previous studies [4,11,44] suggested that manual therapy and needle interventions produce immediate activations of the autonomic nervous system as a result of descendent neurophysiological analgesic pathways of induced analgesia. It is possible that dry needling and manual therapy interventions share underlying mechanisms as previously suggested [9], although further research is now needed.

### 4.5. Limitations

Several limitations in this clinical trial should be acknowledged. First, there are several factors modulating and influencing the autonomic nervous system, such as caffeine and tobacco consumption, psychological conditions (e.g., stress, anxiety, or depression), or the menstrual cycle. Therefore, since these factors were not controlled during the study, our results could be biased. Secondly, the sample size and diversity of the pain population is another limitation of this study. This sample consisted of people seeking physical therapy care with limits of sociodemographic differences and further research is needed considering this suggestion for better generalizability of the results. Finally, larger follow-up periods and a greater number of interventions are needed to better understand long-term adaptative responses of dry needling interventions.

## 5. Conclusions

Our results suggested that dry needling applied to patients with chronic nonspecific neck pain produced an immediate decrease in mechanical hyperalgesia at local sites and produced an increase in skin conductance as compared with sham needling. However, a single session of sham or real dry needling was similarly effective for decreasing related- disability, pain intensity, catastrophism, and kinesiophobia levels seven days after. Further studies are needed to better understand the clinical implications of autonomic nervous system activation on central sensitization and pain processing in the long-term after the application of dry needling.

## Figures and Tables

**Table 1:** Demographic and clinical characteristics of both groups.

	Sham Needling (*n* = 30)	Dry Needling (*n* = 30)	*p* Value
Demographic Data			
Sex (Male/Female)	18/12	14/16	0.301
Weight (kg)	72.3 ± 12.7	68.8 ± 11.8	0.272
Height (m)	1.73 ± 0.09	1.71 ± 0.07	0.187
BMI (kg/m^2^)	23.97 ± 3.02	23.59 ± 3.43	0.649
Age (years)	21.33 ± 2.56	22.70 ± 4.05	0.125
Clinical Data			
Side of intervention (Left/Right)	13/17	13/17	1.000
Duration of symptoms (months)	16.60 ± 9.29	19.13 ± 7.74	0.256
Pain intensity (0–10)	4.93 ± 1.38	5.53 ± 1.59	0.125
NPNPQ (0–100)	17.29 ± 8.74	19.07 ± 9.26	0.446
PCS (0–52)	1.03 ± 0.64	1.26 ± 0.75	0.194
TSK-11 (11–44)	1.07 ± 0.62	1.14 ± 0.60	0.639

Abbreviatures: BMI: Body Mass Index; NPNPQ: Northwick Park Neck Pain Questionnaire; PCS: Pain Catastrophizing Scale; TSK-11: Tampa Scale of Kinesiophobia.

**Table 2:** Skin conductance measurements in both real and sham needling groups.

	Measurement	Sham Needling (*n* = 30)	Dry Needling (*n* = 30)	Within Group Differences (95% CI)		Between Group Differences
				Sham Needling	Dry Needling	
**Mean Skin Conductance**	Pre-Intervention	1.47 (0.95)	1.19 (0.52)	0.68 (0.32; 1.05) *p* < 0.001 ^1^ 1.13 (0.53; 1.74) *p* < 0.001 ^2^ 0.19 (−0.06; 0.44) *p* = 0.269 ^3^	0.60 (0.24; 0.96) *p* < 0.001 ^1^ 1.54 (0.94; 2.15) *p* < 0.001 ^2^ 0.28 (0.03; 0.53) * *p* = 0.022 ^3^	−0.27 (−0.67; 0.13)
	Verbal Warning	2.16 (1.42)	1.80 (0.79)			−0.36 (−0.97; 0.25)
	Intervention	2.60 (1.69)	2.74 (1.42)			0.14 (−0.67; 0.95)
	Post-Intervention	1.66 (1.02)	1.47 (0.72)			−0.18 (−0.64; 0.27)
**Maximum Skin Conductance**	Pre-Intervention	1.54 (1.00)	1.25 (0.57)	1.10(0.56; 1.63) *p* < 0.001 ^1^ 1.28 (0.55; 2.01) *p* < 0.001 ^2^ 0.20 (22120.07; 0.47) *p* = 0.289 ^3^	1.00 (0.47; 1.53) *p* < 0.001 ^1^ 1.88 (1.15; 2.60) *p* < 0.001 ^2^ 0.30 (0.03; 0.57) * *p* = 0.02 ^3^	−0.29 (−0.71; 0.12)
	Verbal Warning	2.64 (1.86)	2.25 (1.10)			−0.39 (−1.18; 0.40)
	Intervention	2.82 (1.92)	3.12 (1.72)			0.30 (−0.64; 1.24)
	Post-Intervention	1.74 (1.13)	1.55 (0.76)			−0.19 (−0.69; 0.30)
**Minimum Skin Conductance**	Pre-Intervention	1.37 (0.91)	1.15 (0.49)	0.18 (0.008; 0.35) *p* = 0.036 ^1^ 0.94 (0.5; 1.37) *p* < 0.001 ^2^ 0.18 (−0.59; 0.41) *p* = 0.269 ^3^	0.26 (0.08; 0.42) *p* = 0.001 ^1^ 1.05 (0.61; 1.49) *p* < 0.001 ^2^ 0.25 (0.02; 0.50) * *p* = 0.03 ^3^	−0.22 (−0.60; 0.16)
	Verbal Warning	1.55 (1.00)	1.41 (0.65)			−0.14 (−0.58; 0.30)
	Intervention	2.30 (1.46)	2.20 (0.99)			−0.10 (−0.75; 0.54)
	Post-Intervention	1.55 (0.93)	1.40 (0.68)			−0.14 (−0.56; 0.28)

^1^ Verbal Warning—Pre-Intervention Difference; ^2^ Intervention—Pre-Intervention Difference; ^3^ Post-Intervention—Pre-Intervention Difference.

**Table 3:** Real and sham dry needling effects on clinical and psychological variables at one-week follow-up.

	Measurement	Sham Needling (*n* = 30)	Dry Needling (*n* = 30)	Within Group Differences (95% CI)		Between Groups Differences
				Sham Needling	Dry Needling	
**Pain Intensity (0−10)**	Before intervention	4.93 ± 1.38	5.53 ± 1.59	−1.73 (−2.46; −0.99) *p* < 0.001	−2.33 (−3.06; −1.59) *p* < 0.001	−0.60 (−1.37; 0.17)
	1-week after	3.20 ± 2.20	3.20 ± 2.02			0.0 (−1.09; 1.09)
**NPNPQ (0−100)**	Before intervention	17.29 ± 8.74	19.07 ± 9.26	−4.42 (−7.25; −1.59) *p* = 0.003	−9.24 (−12.06; −6.41) *p* < 0.001	−1.78 (−6.43; 2.87)
	1-week after	12.86 ± 9.20	9.83 ± 8.10			3.03 (−1.44; 7.51)
**PCS (0−52)**	Before intervention	1.03 ± 0.64	1.26 ± 0.75	−0.28 (−0.50; −0.07) *p* = 0.009	−0.34 (−0.56; −0.13) *p* = 0.002	−0.23 (−0.59; 0.12)
	1-week after	0.74 ± 0.63	0.91 ± 0.83			−0.17 (−0.56; 0.20)
**TSK-11 (11−44)**	Before intervention	1.07 ± 0.62	1.14 ± 0.60	−0.19 (−0.38; −0.06) *p* = 0.044	−0.12 (−0.31; 0.06) *p* = 0.207	−0.07 (−0.38; 0.24)
	1-week after	0.87 ± 0.63	1.01 ± 0.66			−0.14 (−0.48; 0.18)

NPNPQ: Northwick Park Neck Pain Questionnaire; PCS: Pain Catastrophizing Scale; TSK-11: Tampa Scale of Kinesiophobia. Values are mean ± standard deviation and mean [95% Confidence Interval] for differences.

**Table 4:** Psychophysical outcomes, pain pressure thresholds, and quantitative sensory testing differences within and between groups.

	Measurement	Sham Needling (*n* = 30)	Dry Needling (*n* = 30)	Within Group Differences		Between Groups Differences
				Sham Needling	Dry Needling	
PPT C5-6 Homolateral Side (Kg/cm^2^)	Before intervention	3.34 ± 1.28	3.55 ± 1.45	−0.19 (−0.48; 0.08) *p* = 0.167	0.43 (0.15; 0.72) *p* = 0.003 *	−0.20 (−0.91; 0.50)
	Post-Intervention	3.14 ± 1.40	3.98 ± 1.50			−0.83 (−1.59; −0.08) * *p* = 0.03
PPT C5-6 Contralateral Side (Kg/cm^2^)	Before intervention	3.41 ± 1.37	3.55 ± 1.54	0.01 (−0.27; 0.28) *p* = 0.962	0.29 (0.01; 0.57) *p* = 0.038	−0.14 (−0.89; 0.61)
	Post-Intervention	3.42 ± 1.60	3.85 ± 1.52			−0.42 (−1.23; 0.37)
PPT Tibialis Anterior (Kg/cm^2^)	Before intervention	6.21 ± 1.54	6.91 ± 2.56	−0.31 (−0.91; 0.28) *p* = 0.295	−0.25 (−0.85; 0.34) *p* = 0.396	−0.54 (−1.79; 0.39)
	Post-Intervention	5.89 ± 1.92	6.65 ± 3.21			−0.75 (−2.12; 0.61)
PPT Second Finger (Kg/cm^2^)	Before intervention	5.71 ± 2.26	5.95 ± 1.67	−0.12 (−0.63; 0.37) *p* = 0.623	−0.18 (−0.68; 0.31) *p* = 0.453	−0.23 (−1.26; 0.79)
	Post-Intervention	5.59 ± 2.43	5.76 ± 2.20			−0.17 (−1.37; 1.02)
TS difference 1 to 10 stimuli(0−10, NPRS)	Before intervention	−0.33 ± 1.58	−0.50 ± 1.63	0.33 (−0.89; 0.22) *p* = 0.244	0.20 (−0.36; 0.76) *p* = 0.483	−0.17 (0.99; 0.66)
	Post-Intervention	0.00 ± 1.55	−0.30 ± 1.80			−0.30 (−0.57; 1.17)
PPT Difference after and before CPM (Kg/cm^2^)	Before intervention	0.20 ± 1.34	0.13 ± 0.99	0.02 (−0.53; 0.57) *p* = 0.945	0.11 (−0.66; 0.45) *p* = 0.702	−0.07 (−0.68; 0.53)
	Post-Intervention	0.22 ± 1.34	0.24 ± 1.18			0.02 (−0.63; 0.66)
TS 10 stimulus difference with and without CPM (0−10, NPRS)	Before intervention	−0.43 ± 1.33	−0.56 ± 1.54	−0.15 (−0.83; 0.53) *p* = 0.661	0.13 (−0.54; 0.81) *p* = 0.697	−0.13 (−0.87; 0.61)
	Post-Intervention	−0.58 ± 1.11	−0.43 ± 1.45			0.15 (−0.51; 0.81)

CPM: Conditioned Pain Modulation; PPT: Pressure Pain Threshold; TS: Temporal Summation. Values are mean ± standard deviation and mean [95% Confidence Interval] for differences. * Statistically significant differences.

## Data Availability

All data derived from this study are presented in the text.
